# Myofibroblast induces hepatocyte-to-ductal metaplasia via laminin–ɑvβ6 integrin in liver fibrosis

**DOI:** 10.1038/s41419-020-2372-9

**Published:** 2020-03-23

**Authors:** Ting Xu, Zhiwen Lu, Zhuanglong Xiao, Fang Liu, Yuhua Chen, Zhijun Wang, Shenghua Zhu, Yuhu Song

**Affiliations:** 1 Division of Gastroenterology, Union Hospital, Tongji Medical College, Huazhong University of Science and Technology, 430022 Wuhan, China; 2 Institute of Hematology, Union Hospital, Tongji Medical College, Huazhong University of Science and Technology, 430022 Wuhan, China

**Keywords:** Diseases, Stem-cell research

## Abstract

Hepatocytes undergo the metaplasia into ductal biliary epithelial cells (BECs) in response to chronic injury, and subsequently contribute to liver regeneration. The mechanism underlying hepatocyte-to-ductal metaplasia has not been explored until now. In mouse models of liver fibrosis, a florid BEC response was observed in fibrotic liver, and the depletion of myofibroblasts attenuated BEC expansion remarkably. Then, in hepatocyte fate-tracing mouse model, we demonstrated the conversion of mature hepatocytes into ductal BECs in fibrotic liver, and the depletion of myofibroblasts diminished the hepatocyte-to-ductal metaplasia. Finally, the mechanism underlying the metaplasia was investigated. Myofibroblasts secreted laminin-rich extracellular matrix, and then laminin induced hepatocyte-to-ductal metaplasia through ɑvβ6 integrin. Therefore, our results demonstrated myofibroblasts induce the conversion of mature hepatocytes into ductal BECs through laminin-ɑvβ6 integrin, which reveals that the strategy improve regeneration in fibrotic liver through the modification of specific microenvironment.

## Introduction

The liver is a highly regenerative organ with the ability to restore its function after acute injury and chronic injury^1–4^. The cellular sources of regenerative hepatocytes in liver injury is a fundamental issue in liver biology^3–8^. In response to acute injury or loss of liver mass, remaining healthy liver cells proliferate to restore their functions^4,9^. During chronic injuries, liver progenitor cells (LPCs) derived from quiescent facultative stem cells expand, and differentiate to hepatocytes and cholangiocytes^4^. Recently, genetic lineage tracing by labeling a specific type of cells defines the origin of the cells in animal models. Lineage tracing studies have demonstrated that hepatocytes are regenerated by self-replication rather than derived from LPCs or myofibroblasts (MFBs) in chronic liver injuries induced by chemicals^10–16^. Tarlow et al. revealed hepatocytes undergo reversible ductal metaplasia to a distinctive progenitor state, and give rise to hepatocyte nuclear factor (HNF) 4ɑ and Sry HMG box protein 9 (SOX9)-double-positive (HNF4ɑ^+^SOX9^+^) cells during chronic liver injury; HNF4ɑ^+^SOX9^+^ cells differentiate into mature hepatocytes as well as ductal biliary epithelial cells (BECs)^17^. Joan Font-Burgada et al. identified a subpopulation of periportal hepatocytes named as hybrid hepatocytes (HybHP) express low amounts of SOX9 and normal amount of HNF4ɑ. The HybHP make major contribution to parenchymal restoration after chronic liver damage^16^. All these findings suggested mature hepatocytes in periportal area undergo reversible ductal metaplasia to distinctive ductal BECs, which contributes to liver regeneration during chronic injuries. However, the mechanism underlying hepatocyte-to-ductal metaplasia has not been explored until now.

Stem-cell populations are established in niches or specific anatomic locations which maintain and regulate stem cell homeostasis. LPC niche is composed of hepatic stellate cells (HSCs), endothelial cells, macrophages, other inflammatory cells, extracellular matrix (ECM), growth factors, and cytokines^8,18,19^. HSCs, also known as Ito cells, are located in the space of Disse. During chronic liver injury, quiescent HSCs develop into contractile myofibroblast-like cells^20–22^. It is well-known that activated HSCs/MFBs play an important role in liver fibrosis through promoting ECM deposition. Moreover, MFBs in LPC niche are involved in the differentiation of LPC^23–25^. In a choline-deficient ethionine-supplemented (CDE)-induced model of chronic liver injury, ECM deposition and HSC activation occurred as an initial phase, prior to LPC expansion^26^. Inhibition of HSC activation by 2% l-cysteine diminished LPC expansion in animal models^27^. All these revealed a critical role of myofibroblast in LPC expansion. However, the role of MFBs in hepatocyte-to-ductal metaplasia has not been investigated until now. The aim of our study is to investigate the role of MFBs in the conversion of mature hepatocytes into ductal BECs in liver fibrosis.

## Materials and methods

### Animals

C57BL/6 mice were purchased from Beijing Vital River Laboratory Animal Technology Co. Ltd. (Beijing, China). R26R-EYFP mice (*Rosa*26^*loxP-stop-loxP-EYFP*^) were obtained from the Jackson Laboratory (Bar Harbor, ME, USA; 006148). The R26R-EYFP reporter mice contain a loxP-flanked STOP sequence followed by EGFP in the *Rosa*26 locus^28^. All animals were housed in specific pathogen-free (SPF) animal facility. The protocol of animal treatment used in this study was approved by the institutional animal care and use committee of Tongji Medical College, Huazhong University of Science and Technology.

### Animal model of liver fibrosis

Mouse models of liver fibrosis (8 mice per group) were established through the administration of thioacetamide (TAA), carbon tetrachloride (CCl_4_), N-Nitrosodiethylamine (DEN), and tetrachloride (CCl_4_). Chemicals used were listed in Supplementary Table 1

### TAA administration

Male mice (6–8 weeks old) were treated three times a week intraperitoneal (i.p.) injections of 150 mg/kg TAA for 6 weeks.

### CCl_4_ administration

Male mice (6–8 weeks old) were injected subcutaneously with CCl_4_ diluted 5:5 (v/v) ratio in olive oil at a dose of 3 ml/kg twice a week for 12 weeks^21^.

### The administration of DEN and CCl_4_

15-day-old mice were injected intraperitoneally (i.p.) with DEN at a dose of 25 mg/kg. At 29 days, mice were injected intraperitoneally with CCl_4_ diluted 1:9 (v/v) ratio in olive oil at the dose of 5 ml/kg weekly for 12 weeks^29,30^.

### Lineage-tracing of mature hepatocytes using AAV infection

For lineage-tracing of mature hepatocytes, the R26R-EYFP reporter mice (*Rosa*^YFP^) were injected with adeno-associated virus-thyroxine-binding globulin (TBG)-Cre^11,14,29^. Recombinant adeno-associated virus 2/8 (AAV2/8) exhibits hepatocyte-specific tropism. AAV8-TBG-Cre contains a liver-specific promoter (TBG promoter) driving Cre. Replication-incompetent AAV2/8-TBG-Cre virus (AAV-TBG -Cre) carrying Cre expression under the control of TBG promoter was packaged and purified by Biowit biotechnologies (Shenzhen, China)^31^. Two protocols were applied in lineage-tracing of mature hepatocyte. 6-day-old *Rosa*^YFP^ mice were injected intraperitoneally with AAV8-TBG-Cre (4 × 10^10^ genome copies per mouse). The *Rosa*^YFP^ mice (4–6 weeks old) were injected intravenously with AAV8-TBG-Cre (2.5 × 10^11^ genome copies of virus per mouse).

### Depletion of MFBs by DAPT in mouse models of liver fibrosis

To deplete MFBs, N-[N-(3,5-difluorophenacetyl)-l-alanyl]-S-phenylglycine t-butylester (DAPT, Table S1), a γ-secretase inhibitor, was administrated into mouse models of liver fibrosis^25,32^. For CCl_4_ and DEN/CCl_4_-induced liver fibrosis, the mice received antifibrotic treatment with DAPT (50 mg/kg) after 8-week exposure of CCl_4_ by intraperitoneal injection five times a week for another 4 weeks. For TAA-induced liver fibrosis, the mice received antifibrotic treatment with DAPT (50 mg/kg) after 2-week exposure of TAA by intraperitoneal injection five times a week for another 4 weeks.

### Immunohistochemistry and immunofluorescent staining

The sections of formalin-fixed, paraffin-embedded liver samples were stained with hematoxylin and eosin for standard histology. For the assessment of collagen deposition, Sirius Red staining was performed using the staining assay kit according to the manufacturer’s instructions. For immunohistochemical staining or immunofluorescent staining, the slides were incubated with primary antibodies (Table S1) followed by appropriate secondary antibodies (Table S1). For immunofluorescent staining,the slides were mounted with DAPI-containing medium and the images were acquired with a Nikon-A1-si confocal microscope.

### HSC isolation

Mouse HSCs were obtained by in situ perfusion with collagenase type IV, pronase E, and DNAase followed by differential centrifugation on Opti-Prep density gradients^21^. Cell viability was assessed by trypan blue exclusion. Primary HSCs were cultured in DMEM/10% fetal bovine serum (FBS) containing penicillin and streptomycin. The purity of activated HSCs (MFBs) was revealed by immunofluorescence using anti-ɑ-smooth muscle actin (ɑSMA) antibody.

### Hepatocyte isolation

Hepatocytes were isolated by a two-step perfusion technique. Mouse hepatocytes were isolated from the digested liver by centrifugation^33^. Cell viability was assessed by trypan blue exclusion. Primary hepatocytes were cultured in DMEM/10% FBS containing penicillin and streptomycin. The purity of isolated hepatocytes was demonstrated by immunofluorescence using anti-albumin antibody.

### Flow cytometry analysis

Liver nonparenchymal cells (NPCs) were isolated by a multistep collagenase perfusion and the incubation with the antibodies as previously described^17,34^. In brief, dissociated cells were incubated with APC-conjugated anti-mouse MIC1-1C3 antibody, Percp cy5.5-conjugated anti-mouse CD26 antibody, PE-cy7-conjugated anti-mouse CD11b, PE-cy7-conjugated anti-mouse CD31, and PE-cy7-conjugated anti-mouse CD45 antibody (Table 1) at 4 °C for 30 min. Then liver NPCs were incubated with PE-conjugated PI at room temperature for 5 min. Finally, the samples were ready for analysis with LSRFortessa (BD).

**Table 1:** Key resourse table.

Reagents or resources	Source	Application	Catalog number
*Antibodies*			
Rabbit anti-HNF4α	Abcam	IHC	ab201460
Rabbit anti-CK19	Proteintech	IHC, IF	10712-1-AP
Rabbit anti-SOX9	Millipore	IHC, IF	AB5535
Goat anti-OPN	R&D systems	IHC, IF	AF808
Rabbit anti-GFP	Proteintech	IF	50430-2-AP
Rabbit anti-aSMA	Abcam	IHC, IF	ab5694
Rabbit anti-CD31	Abcam	IHC	ab28364
Goat anti-GFP	Abcam	IF	ab6673
Rat anti-F4/80	Abcam	IHC	ab6640
Goat anti-ITGB6	R&D systems	IHC, IF	AF2389
Rabbit anti-ITGB6	ABclonal technology	WB	A16904
Rabbit anti-Alb	Proteintech	IF	16475-1-AP
Rabbit anti-HNF4α	ArigoBiolaboratories	IF	ARG-55328
Rabbit anti-Laminin	Abcam	IP	ab11575
Rat anti-MIC1-13	Grompe Lab	Flow cytometry	Gift
APC Rat anti-mouse MIC1-1C3	BD PharMingen	Flow cytometry	NBP1-18961
PE-Cy7 Rat anti-mouse CD11b	BD PharMingen	Flow cytometry	552850
PE-Cy7 Rat anti-mouse CD31	BD PharMingen	Flow cytometry	561410
PE-Cy7 Rat anti-mouse CD45	BD PharMingen	Flow cytometry	552848
Percp-Cyanine5.5 anti-mouse CD26	eBioscience	Flow cytometry	H194-112
Alexa Fluor 647 Goat anti Rat IgG (H + L)	Cell Signaling Technology	Flow cytometry	4418S
Alexa Fluor 488 Donkey anti Rabbit IgG (H + L)	Antgene	IF	ANT024s
Alexa Fluor 594 Donkey anti Rabbit IgG (H + L)	Antgene	IF	ANT030s
Alexa Fluor 594 Donkey anti Rabbit IgG (H + L)	Life technologies	IF	A21207
Alexa Fluor 488 Donkey anti goat IgG (H + L)	Antgene	IF	ANT025
Alexa Fluor 594 Donkey anti goat IgG (H + L)	Antgene	IF	ANT031
*Chemicals, peptides, and recombinant proteins*			
Thioacetamide(TAA)	TCI	Mice model	T0187
N-Nitrosodiethylamine (DEN)	TCI	Mice model	D0516
Carbon tetrachloride	Makclin	Mice model	C822982
Olive oil	Makclin	Solvent for CCl_4_	O815211
Corn oil	Sigma	Solvent for DMSO	C8267
DAPT (GSI-IX)	Selleck&Bimake	Depletion of myofibroblast	S2215
Dimethyl sulfoxide (DMSO)	MP Biomedicals	Solvent	196055
Laminin	Sigma	Cell culture	L2020
Penicillin–streptomycin	Sigma	Cell culture	V900929
DMEM basic	Gibco	Cell culture	C11995500BT
Fetal bovine serum	Gibco	Cell culture	10270-106
Sirius red staining	Solarbio	ECM depostion	G1470-2
DAPI	Sigma	IF	28718-90-3
Propidium iodide	Sigma	Flow cytometry	P4170
Trizol reagent	Invitrogen	RNA extraction	15596-018
RNAiso Plus	Takara	RNA extraction	9109,
TB Green™ Premix Ex Taq™	Takara	RT-PCR	RR420A
PrimeScript™ RT Master Mix	Takara	RT	RR036A
Collagenase Type IV	Gibco	HSC isolation	9001-12-1
DNase I	Roche	HSCisolation	10104159001
Lipofectamine^TM^2000	Invitrogen	Cell transfection	11668-019
*Software and algorithms*			
Adobe Photoshop CS6	Adobe	Photo	Version19.1.2
NIS-Elementsviewer	Laboratory Imaging	IF	Version3.20.02
ImageJ	National Institutes of Health	IHC	Version1.51j8
GraphPad prism	GraphPad Software	Photo	Version5.0.1
FlowJo	Becton, Dickinson & Company	Flow cytometry	Version10.0.7

### Co-culture of MFBs and hepatocytes

To determine the effect of MFBs on hepatocytes, MFBs were co-cultured with hepatocytes using cell culture inserts (0.3 µm pore size). In brief, MFBs were seeded in the upper chamber, and hepatocytes isolated from normal or fibrotic liver were grown in the bottom chamber. To determine the effect of laminin on biological characteristic of hepatocytes, hepatocytes from fibrotic liver were grown in laminin-coated culture plate. In all, 60 µg/ml laminin was added to culture plates and maintained at a final concentration of 3 μg/cm^2^ at 37 °C for 2 h, or blow-dried on a clean bench at room temperature overnight.

### qRT-PCR

Total RNA was isolated using Trizol reagent, and 10 µg of total RNA was used for cDNA synthesis, using an RT kit. Real-time polymerase chain reaction (PCR) was performed using Taraka TB Green™ Premix Ex Taq™ (Table S1). Reactions were performed twice in triplicate. Expression was normalized to GAPDH and quantified using the 2^–ΔΔCt^method. The following primers were used:

*Gapdh* (XM_017321385.1) Forward: 5′-AGGTCGGTGTGAACGGATTTG-3′,

Reverse: 5′-TGTAGACCATGTAGTTGAGGTCA-3′,

*Itgb6* (XM_006498810.3) Forward: 5′-CAACTATCGGCCAACTCATTGA-3′,

Reverse: 5′-GCAGTTCTTCATAAGCGGAGAT-3′.

### RNA-sequencing

Total RNA was extracted from hepatocytes using TRIzol reagent following the manufacturer’s instructions. The Libraries were generated using the VAHTS Stranded mRNA-seq Library Prep Kit for Illumina® (Vazyme), and were subsequently sequenced by an Illumina HiSeq X-ten. RNA isolation, library construction, and sequencing were performed at Shanghai Biotechnology Corporation (Shanghai, China). For data analysis, the raw reads were filtered by Seqtk before mapping to genome using Tophat (version: 2.0.9). The fragments of genes were counted using HTSeq. Significant differential expressed genes (DEGs) were identified as those with a false discovery rate (FDR) value above the threshold (*Q* < 0.05) and fold-change >2 using edgeR software.

### Immunoprecipitation and Western blot

Lysates from cells and tissues were collected using RIPA buffer (Sigma R0278, St Louis, MO, USA), and then immunoprecipitated with primary antibodies (Table S1). Equal amounts of protein was separated on SDS–polyacrylamide gels, immunoblotted with primary antibodies, then with horseradish peroxidase-conjugated secondary antibodies. The blot was washed three times and was developed with ECL according to the manufacturer’s instructions. Antibodies used are listed in Table 1.

### siRNA transfection

Chemically synthesized siRNAs and the controls were transfected into primary hepatocytes by Lipofectamine^TM^2000 in accordance with the manufacturer’s directions. 48–72 h after transfection, transfected cells were collected for further study.

### Statistical analysis

Data are expressed as mean ± SEM. Comparisons between two groups were made by Student’s two tailed *t*-tests. *P* < 0.05 was considered significant. Statistics and graphing were performed using Prism 5.0.1 (GraphPad) software. All experiments were analyzed from *n* ≥ 3 independent experiments.

## Results

### Inhibition of HSC activation diminishes the expansion of ductal BECs

**Fig. 1:**
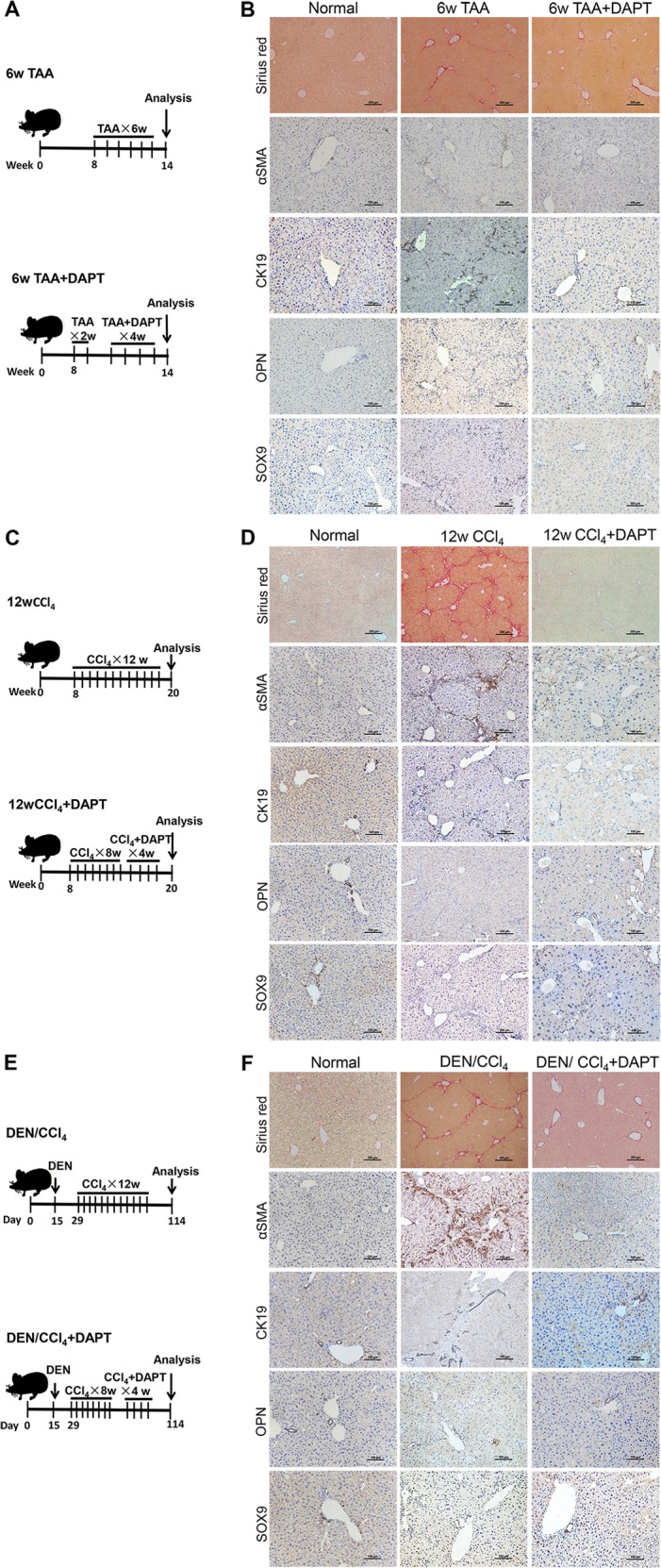
Inhibition of HSC activation diminished the expansion of ductal biliary epithelial cells (BECs). **a** Experiment design for TAA-induced liver fibrosis and DAPT-mediated inhibition of HSC activation in vivo. Vertical lines represent weekly intraperitoneal injections of TAA or TAA/DAPT. TAA thioacetamide, DAPT N-[N-(3,5-difluorophenacetyl)-l-alanyl]-S-phenylglycinet-butylester. **b** Representative liver sections from TAA-treated mice administrated with DAPT or the control (Sirius red staining, immunohistochemical staining). Collagen deposition was determined by Sirius red staining, immunohistochemical staining showed the expression of myofibroblasts (ɑSMA) and ductal BECs (CK19, OPN, and SOX9). **c** Experiment design for CCl_4_-induced liver fibrosis and DAPT-mediated inhibition of HSC activation in vivo. **d** Representative liver sections from CCl_4_-treated mice administrated with DAPT or the control (Sirius red staining, immunohistochemical staining). **e** Experiment design for DEN/CCl_4_-induced liver fibrosis and DAPT-mediated inhibition of HSC activation in vivo. DEN N-Nitrosodiethylamine. **f** Representative liver sections from DEN/CCl_4_-treated mice administrated with DAPT or the control (Sirius red staining, immunohistochemical staining).

To determine the role of MFBs/activated HSCs in the expansion of ductal BECs in liver fibrosis, the correlation between HSC activation and ductal BEC expansion was firstly investigated. In mouse model of TAA-induced liver fibrosis, an increased deposition of ECM was demonstrated by Sirius red staining (Figs. 1b and S1), and the conversion of quiescent HSCs into MFBs was revealed by immunostaining of ɑ-SMA (Figs. 1b and S1). Together with ECM deposition, the expansion of ductal BECs was revealed by immunostaining of CK19, SOX9, and OPN (Figs. 1b and S1). All these indicated the infiltration of ductal BECs into liver parenchyma was chaperoned by HSC activation. Furthermore, mouse models of liver fibrosis induced by CCl_4_ and DEN/CCl_4_ were also created, and the results (Figs. 1c–f and S1) demonstrated positive correlation between HSC activation and the expansion of ductal BECs. All these revealed that the expansion of ductal BECs was chaperoned by HSC activation in fibrotic liver. Previous study demonstrate that ECM deposition and activation of matrix-producing cells occurred as an initial phase, prior to LPC expansion^26^. Thus, the role of MFBs in the expansion of ductal BECs was determined in liver fibrosis. Animal models of liver fibrosis were administrated with N-[N-(3,5-difluorophenacetyl)-l-alanyl]-S-phenylglycinet-butylester (DAPT), a γ-secretase inhibitor. Inhibition of γ-secretase resulted in the depletion of MFBs and attenuated liver fibrosis, which was revealed by Sirius red staining and ɑ-SMA expression (Figs. 1b, d, f and S1). In addition, immunofluorescene of ɑ-SMA demonstrated that DAPT inhibited HSCs activation in vitro (Fig. S2). Fortunately, DAPT did not change the expression of endothelial cell (CD 31), macrophage (F4/80), and T lymphocytes (CD4, CD8) (Fig. S3). Importantly, primary hepatocytes isolated from fibrotic liver were treated with DAPT, and the result demonstrated DAPT had no effect on the expression of LPC markers in chronic injured hepatocytes (Fig. S2). All these confirmed the cellular specificity of DAPT-mediated inactivation of MFBs in fibrotic liver. After attenuating liver fibrosis, the mice exhibited a blunted response of ductal BECs accompanied by the inhibition of HSC activation. All these revealed that HSC inactivation diminished the expansion of ductal BECs.

### MFBs induce the conversion of mature hepatocytes into ductal BECs in vivo

**Fig. 2:**
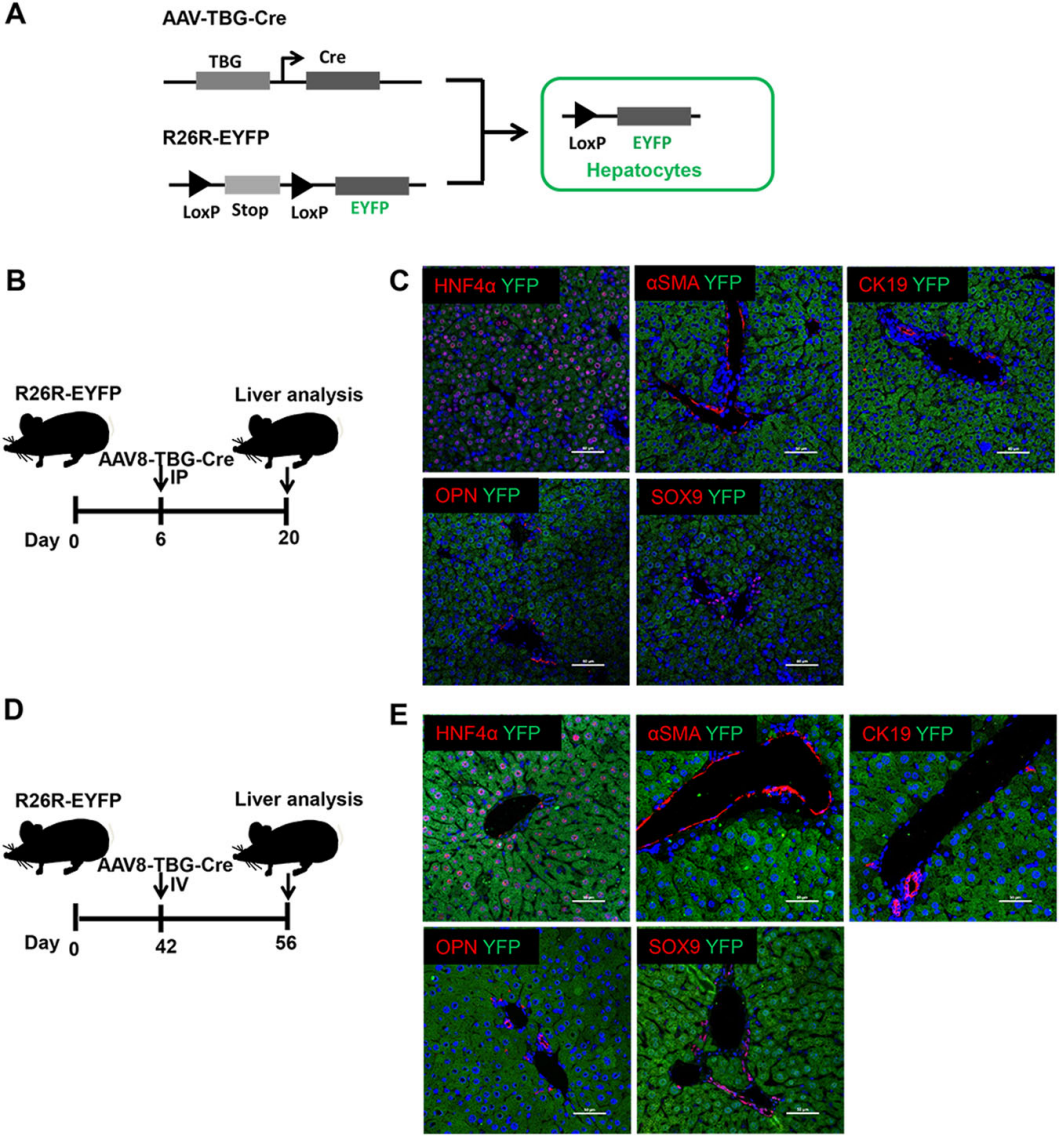
Lineage-tracing of mature hepatocytes through the administration of AAV virus into the R26R-EYFP reporter mice. **a** Schematic diagram of hepatocyte fate-tracing strategy through the injection of AAV8-TBG-Cre into R26R-EYFP (*Rosa*^YFP^) mice. **b** Experiment design for lineage-tracing of mature hepatocytes in 6-day-old *Rosa*^YFP^ mice. 6-day-old *Rosa*^YFP^ mice were intraperitoneally injected with AAV8-TBG-Cre. **c** Immunofluorescence staining demonstrated AAV8-TBG-Cre efficiently labeled mature hepatocytes in 6-day-old *Rosa*^YFP^ mice. **d** Experiment design for lineage-tracing of mature hepatocytes in 6-week-old *Rosa*^YFP^ mice. *Rosa*^YFP^ mice (6-week-old) were intravenously injected with AAV8-TBG-Cre. **e** Immunofluorescence staining demonstrated AAV8-TBG-Cre efficiently labeled hepatocytes in 6-week-old *Rosa*^YFP^ mice.

To determine the conversion of mature hepatocytes into ductal BECs in fibrotic liver, the fate of mature hepatocytes should be tracked specifically in mouse models of liver fibrosis. For lineage-tracing of mature hepatocytes, the R26R-EYFP reporter mice were injected with AAV8-TBG-Cre. In Rosa^YFP^ mice, the expression of YFP is blocked by transcriptional stop sequences flanked by loxP sites. Since the injection of AAV serotype with a high tropism (AAV2/8) for hepatocytes contained the hepatocyte-specific TBG promoter, AAV8-TBG-Cre–mediated loop out of the floxed stop codon resulted in efficient YFP expression in nearly all hepatocytes (Fig. 2). Other hepatic cells, MFBs (ɑSMA+), cholangiocytes (CK19+, SOX9+, OPN+) remained YFP-negative, revealing the specificity of this labeling strategy (Fig. 2).

**Fig. 3:**
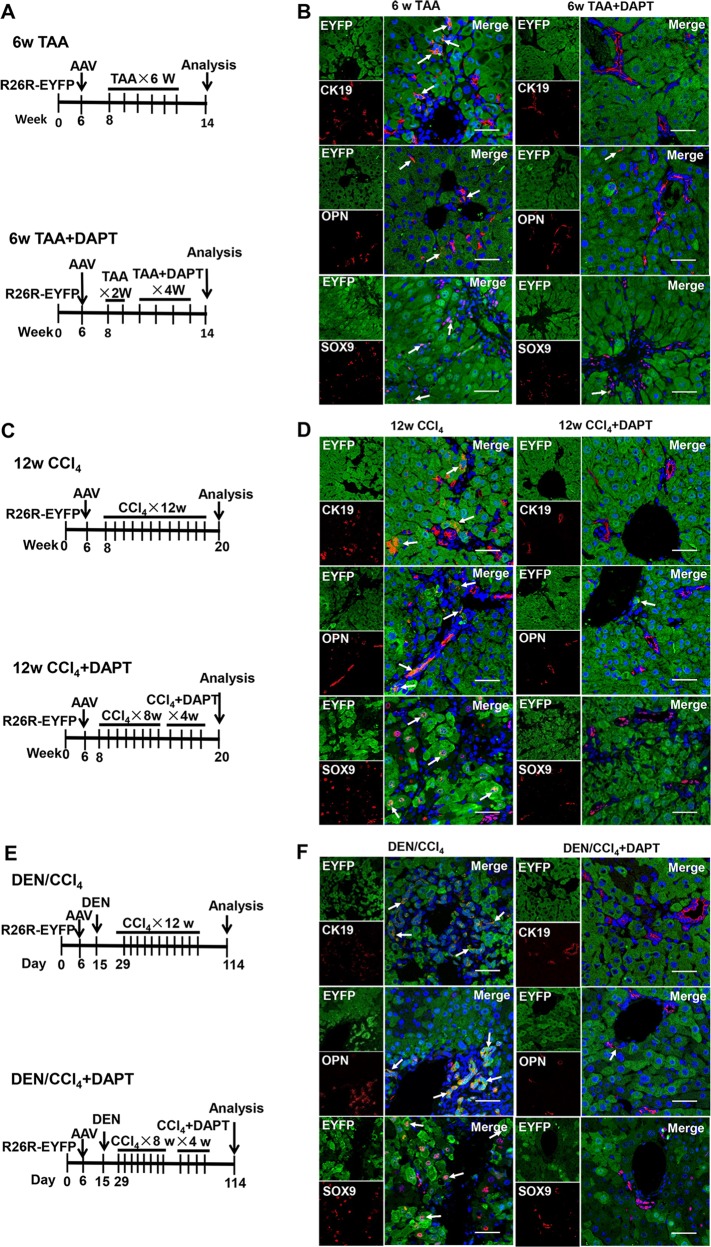
Myofibroblasts induce the conversion of mature hepatocytes into ductal biliary epithelial cells in vivo. **a** Experiment design for DAPT-mediated inhibition of HSC activation in TAA-treated *Rosa*^YFP^ reporter mice. **b** Immunofluorescent staining of fibrotic liver from TAA-treated *Rosa*^YFP^ reporter mice. Immunostaining of ductal BECs marker (CK19, OPN, and SOX9) in liver of *Rosa*^YFP^ mice showed that the percentage of hepatocytes-derived ductal BECs was diminished after DAPT-mediated inhibition of HSC activation in TAA-treated mice. **c** Experiment design for DAPT-mediated inhibition of HSC activation in CCl_4_-treated *Rosa*^YFP^ reporter mice. **d** Immunofluorescence staining of fibrotic liver from CCl_4_-treated *Rosa*^YFP^ reporter mice. **e** Experiment design for DAPT-mediated inhibition of HSC activation in DEN/CCl_4_-treated *Rosa*^YFP^ reporter mice. **f** Immunofluorescence staining of fibrotic liver from DEN/CCl_4_-treated *Rosa*^YFP^ reporter mice. Magnification, ×600.

**Fig. 4:**
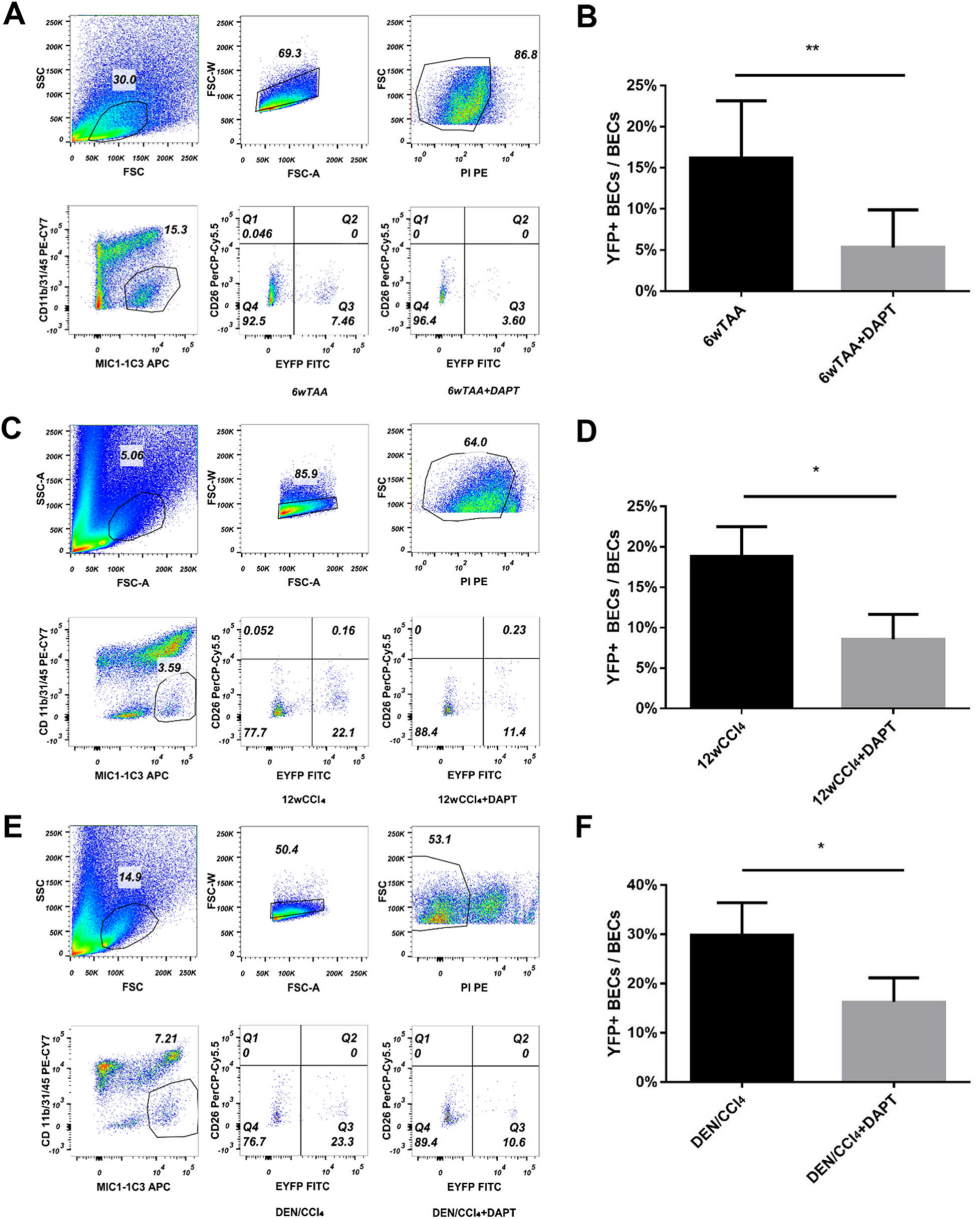
Flow cytometry analysis demonstrated the inhibition of HSC activation diminished the conversion of mature hepatocytes into ductal BECs in vivo. **a** Flow cytometry analysis showed hepatocyte-derived ductal BECs diminished in TAA-treated *Rosa*^YFP^ reporter mice upon DAPT-mediated inhibition of HSC activation. MIC1-1C3 was used as a surface marker of ductal BECs. **b** The percentage of ductal BECs derived from YFP-marked hepatocytes decreased significantly in TAA-treated *Rosa*^YFP^ reporter mice upon DAPT treatment. **c** Flow cytometry analysis showed hepatocyte-derived ductal BECs diminished in CCl_4_-treated *Rosa*^YFP^ reporter mice upon DAPT-mediate inhibition of HSC activation. **d** The percentage of ductal BECs derived from YFP-marked hepatocytes decreased significantly in CCl_4_-treated *Rosa*^YFP^ reporter mice upon DAPT treatment. **e** Flow cytometry analysis showed hepatocyte-derived ductal BECs diminished in DEN/CCl_4_-treated *Rosa*^YFP^ reporter mice upon DAPT-mediate inhibition of HSC activation. **f** The percentage of ductal BECs derived from YFP-marked hepatocytes decreased significantly in DEN/CCl_4_-treated *Rosa*^YFP^ reporter mice after the treatment of DAPT. Each bar represents the mean ± SD for at least triplicate experiments and the *P*-value was determined by Student’s *t*-test (***P < 0.001, **P < 0.01, *P < 0.05).

To determine the effect of MFBs on ductal metaplasia of hepatocytes into ductal BECs, the models of liver fibrosis were established using the R26R-EYFP mice. Firstly, 6-week-old mice were administrated with AAV8-TBG-Cre followed by intraperitoneal injection of TAA (Fig. 3a). In fibrotic liver, some of ductal BECs (labeled as CK19+, SOX9+, OPN+) expressed YFP, indicating that these ductal BECs were derived from mature hepatocytes (Fig. 3b). Secondly, immunostaining of ductal BECs marker (CK19) and MFBs (aSMA) was performed in fibrotic liver of *Rosa*^YFP^ mice. The result (Fig. S4) revealed neighborhood of MFBs and hepacytes-derived ductal BECs. Thirdly, mouse model of TAA-induced liver fibrosis were treated with DAPT. Interestingly, the percentage of YFP-positive BECs (YFP^+^/CK19^+^, YFP^+^/SOX9^+^, YFP^+^/OPN^+^) in BECs (CK19+, SOX9+, OPN+) was reduced in DAPT-treated mice compared with the controls (Figs. 3b and S4B). All these revealed that ductal metaplasia of hepatocytes into ductal BECs was diminished after the depletion of MFBs. To confirm this finding, a flow cytometry-based assay was performed to determine hepatocyte-derived ductal BECs. Ductal BECs were isolated with surface marker MIC1-1C3^14,35^, and the results (Fig. 4a) demonstrated 16.16 ± 1.94% of BECs (MIC1-1C3+) were YFP+ (hepatocyte-derived ductal BECs) after 6 weeks of injury; while in the DAPT-treated group, 5.33 ± 1.61% of BECs (MIC1-1C3+) were YFP+. All these revealed that MFBs induced the conversion of hepatocytes into ductal BECs in fibrotic liver. To further confirm the findings, hepatocyte-derived ductal BECs were analyzed in CCl_4_ and DEN/CCl_4_-induced liver fibrosis using immunofluorescene and flow cytometry. In CCl_4_ or DEN/CCl_4_-induced liver fibrosis, co-immunofluorescene of YFP with ductal BECs markers (CK19, SOX9, OPN) demonstrated ductal metaplasia of hepatocytes into ductal BECs was diminished in DAPT-treated mice (Figs. 3d, f and S4B). In addition, the result of flow cytometry revealed that ductal metaplasia of hepatocytes into ductal BECs was diminished in DAPT-treated mice (Fig. 4c–f). These results indicated that the effect of DAPT on hepatocyte-to-ductal metaplasia resulted from the depletion of MFBs. In summary, all these indicated MFBs contributed to the conversion of hepatocytes into ductal BECs in cirrhotic liver.

### MFBs induce the conversion of mature hepatocytes into ductal BECs in vitro

**Fig. 5:**
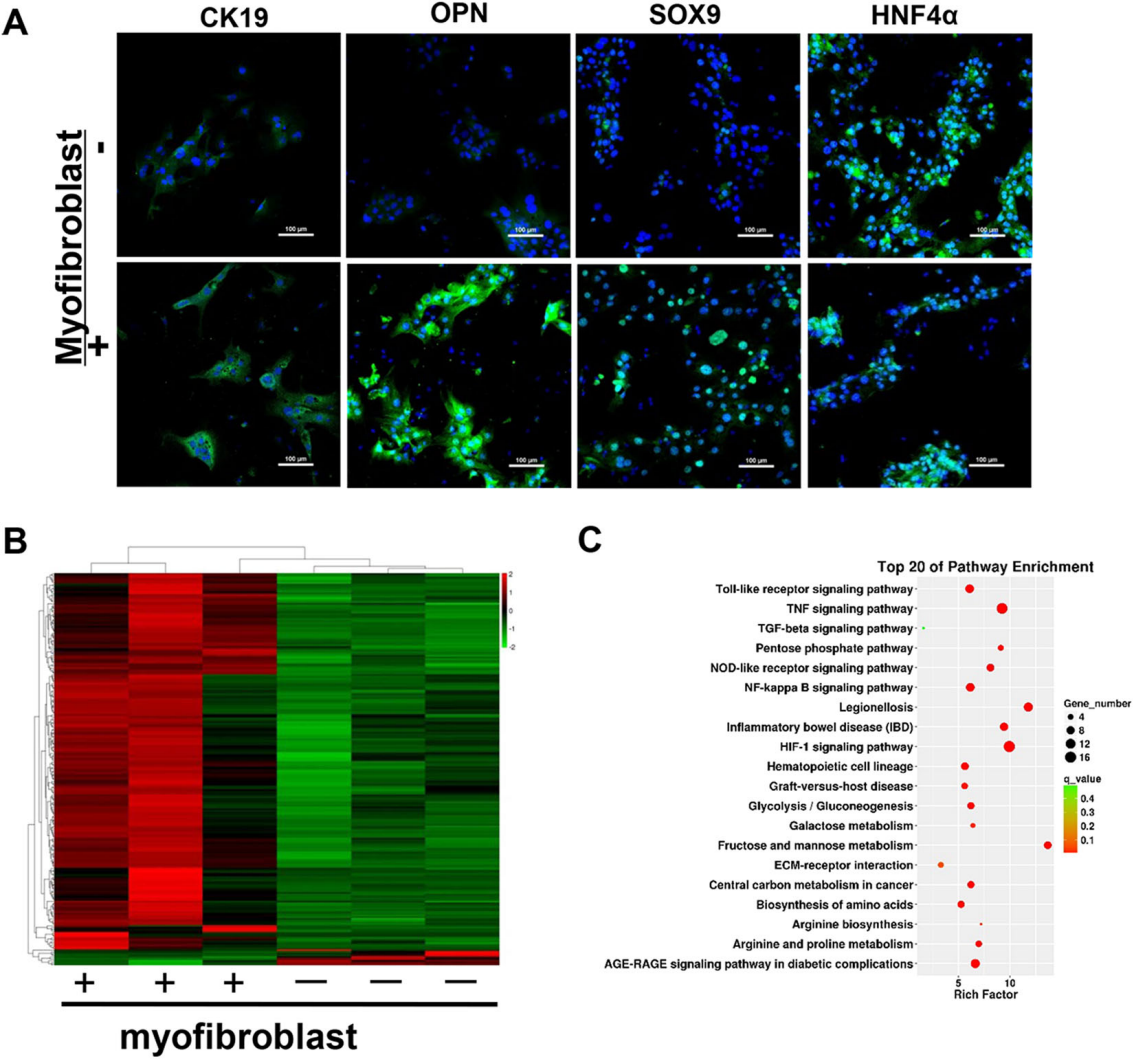
Myofibroblasts induce the conversion of mature hepatocytes into ductal biliary epithelial cells in vitro through co-culture of myofibroblasts and chronic injured hepatocytes. Immunofluorescence staining and RNA-sequencing were performed to determine the effect of myofibroblasts on biological characteristic of chronic injured hepatocytes. **a** Immunofluorescence staining showed the expression of ductal BECs marker increased in chronic injured hepatocytes upon the treatment of myofibroblasts. **b** Heat map of the differentially expressed mRNAs in chronic injured hepatocytes treated with myofibroblasts. **c** Pathway enrichment analysis was performed to identify pathways involved in the conversion of mature hepatocytes into ductal biliary epithelial cells.

To confirm the contribution of MFBs in the conversion of hepatocytes into ductal BECs, the effect of MFBs on biological characteristic of hepatocytes was determined. Firstly, MFBs were co-cultured with hepatocytes isolated from normal mice. Upon the treatment of MFBs, up-regulation of ductal BEC markers (CK19, SOX9, OPN) was observed in hepatocytes (Fig. S5A). Secondly, MFBs were co-cultured with hepatocytes treated with CCl_4_ in vitro, and the results (Fig. S5B) showed similar phenotypic change in hepatocytes. Thirdly, chronic injured hepatocytes were co-cultured with MFBs since chronic injured hepatocytes isolated from fibrotic liver resembled pathological status. Upon the treatment of MFBs, up-regulation of ductal BEC markers was observed in chronic injured hepatocytes (Fig. 5). All these results demonstrate the contribution of MFBs in the conversion of hepatocytes into ductal BECs.

To further explore the mechanism underlying the contribution of MFBs in the conversion of hepatocytes into ductal BECs, chronic injured hepatocytes were subjected to the analysis of RNA-sequencing. In chronic injured hepatocytes co-cultured with MFBs, a total of 216 genes were upregulated and 10 genes were downregulated (Fig. 5b). Then, pathway enrichment analysis was performed to identify pathways that were differentially active between cell subpopulations. As shown in Fig. 5c, gene sets for Toll-like receptor signaling pathway, TNF signaling pathway, TGF-β signaling pathway, NF-Kappa β signaling pathway were significantly induced. During the process of fibrogenesis, HSC activation leads to accumulation of ECM. Importantly, we found gene sets for ECM receptor interaction (laminin and integrin) were induced (Fig. 5c).

### MFBs induce the conversion of mature hepatocytes into ductal BECs through the interaction of laminin-ɑvβ6 integrin

**Fig. 6:**
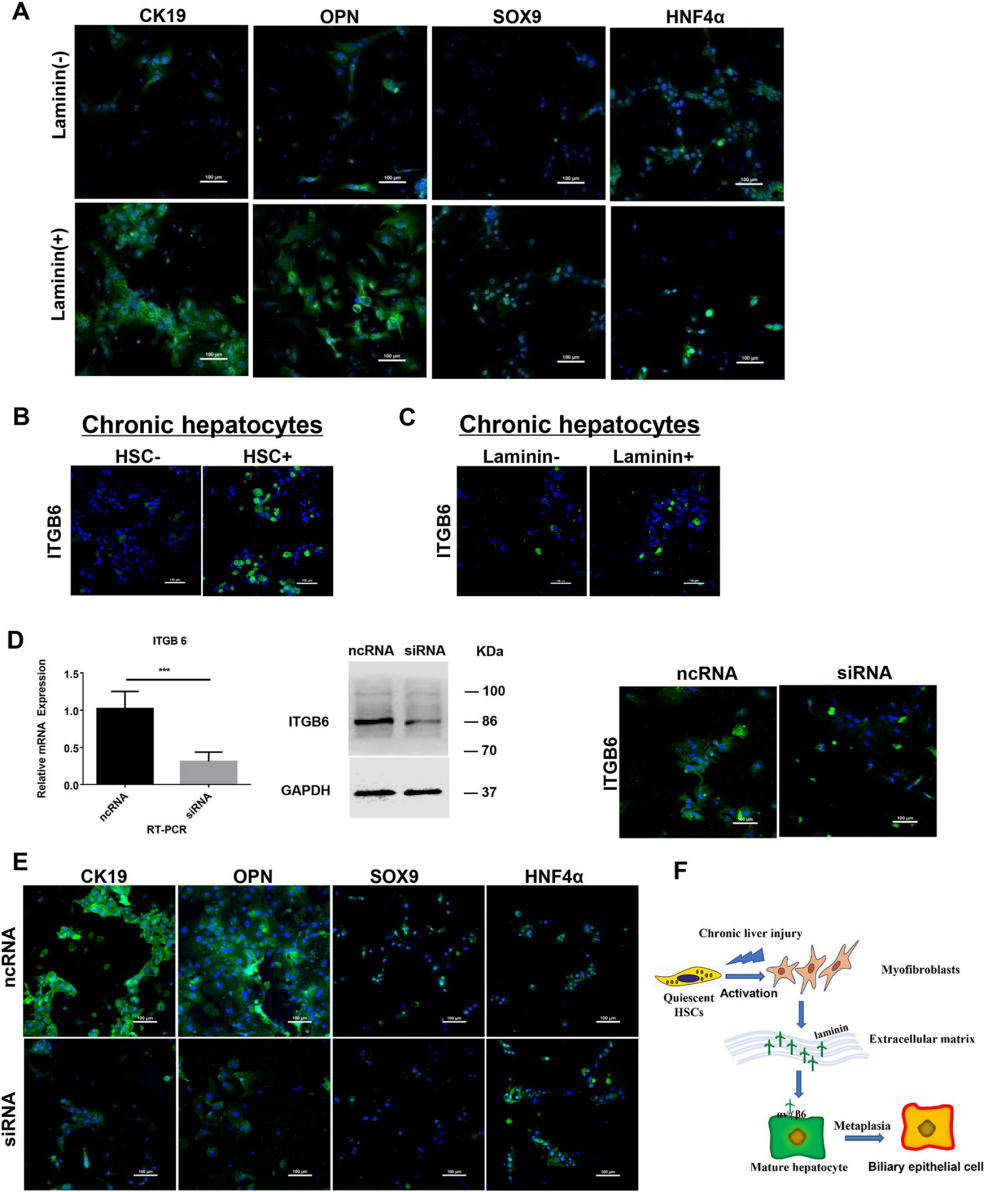
Myofibroblasts induce the conversion of mature hepatocytes into ductal biliary epithelial cells through the interaction of laminin–β6 integrin. **a** Up-regulation of ductal BECs marker (CK19, OPN, and SOX9) and down-regulation of hepatocytes marker (HNF4ɑ) were observed in chronic injured hepatocytes upon the treatment of laminin. **b** Up-regulation of integrin ɑvβ6 expression in chronic injured hepatocytes upon the treatment of myofibroblasts. **c** Up-regulation of integrin ɑvβ6 expression in chronic injured hepatocytes upon the treatment of laminin. **d** siRNA-mediated inhibition of integrin ɑvβ6 expression in chronic injured hepatocytes. Primary hepatocytes isolated from fibrotic liver were transfected with 50 nM of ITGB6-siRNA and control scrambled siRNA (NC), qRT-PCR, western Blot and IF staining demonstrated efficient knockdown of ITGB6 expression through siRNA. **e** Immunofluorescence staining showed down-regulation of ductal BECs markers (CK19, OPN, and SOX9) expression and up-regulation of HNF4ɑ expression in primary hepatocytes transfected with ITGB6-siRNA compared with the control. **f** Graphical summary of signaling pathway in which myofibroblasts induce the conversion of mature hepatocytes into LPCs through the interaction of laminin–integrin ɑvβ6. Each bar represents the mean ± SD for at least triplicate experiments and the *P*-value was determined by Student’s *t*-test (***P < 0.001, **P < 0.01, *P < 0.05).

MFBs are the major source of ECM in fibrotic liver. The extracellular component of the LPC niche are rich in laminin matrix. Previous study demonstrated that laminin deposition was likely to be important prerequisites to LPCs activation and expansion^36^. Thus, we hypothesized that MFBs participated in hepatocyte-to-ductal metaplasia through laminin. To verify the hypothesis, the concentration of laminin was determined and the increase of laminin concentration was observed in culture medium of MFBs compared with the control (Fig. S6A). Additionally, a decrease in laminin concentration was observed after DAPT-mediated depletion of MFBs in vitro (Fig. S6A). Then the effect of laminin on chronic injured hepatocytes was determined. Primary hepatocytes isolated from fibrotic liver were grown on the plates containing laminin, and up-regulation of ductal BECs markers (CK19, OPN, and SOX9) and down-regulation of HNF4ɑ were observed (Figs. 6a and S6B). These indicated that MFBs were involved in the metaplasia of mature hepatocytes into ductal BECs via the secretion of laminin-rich ECM.

Integrin ɑvβ6 is expressed on ductal BECs and critically regulates their function in vivo and in vitro^37,38^. Thus, we hypothesized that MFBs participated in hepatocyte to-ductal metaplasia through the interaction of laminin and integrin ɑvβ6. To test the hypothesis, the effect of integrin ɑvβ6 on the behavior of chronic injured hepatocytes was determined. Firstly, the expression of integrin ɑvβ6 was examined in fibrotic liver. As shown in Fig. S6C, up-regulation of integrin ɑvβ6 expression was observed in periportal area of cirrhotic liver; then immunofluorescence of liver sections demonstrated ductal BECs (CK19+) expressed integrin ɑvβ6 in fibrotic liver (Fig. S6D). To determine the involvement of integrin ɑvβ6 in ductal metaplasia of hepatocytes into ductal BECs, the expression of integrin ɑvβ6 was firstly determined in chronic injured hepatocytes upon the treatment of myofibroblast or laminin. After the treatment of MFBs or laminin, up-regulation of integrin ɑvβ6 expression was observed in chronic injured hepatocytes (Fig. 6b, c). To further determine the involvement of myofibroblast in ductal metaplasia through integrin ɑvβ6, ITGB6-siRNA was transfected into hepatocytes and the effect of integrin ɑvβ6 on the behavior of chronic injured hepatocytes were evaluated. qRT-PCR, western blot, and immunofluorescence showed siRNA suppressed ITGB6 expression efficiently after siRNA transfection (Fig. 6d). After efficient knockdown of integrin ɑvβ6 expression, immunofluorescence staining showed down-regulation of ductal BECs markers (CK19, OPN, and SOX9) and up-regulation of HNF4ɑ in chronic injured hepatocytes (Fig. 6e). To determine laminin induce the hepatocyte-to-ductal metaplasia by binding to integrin, co-immunoprecipitation was performed. The results (Fig. S6E) demonstrated laminins exerted their effect through binding of integrin β6. In summary, MFBs induced the metaplasia of mature hepatocytes into ductal BECs via laminin-ITGB6 signal pathway during the process of liver cirrhosis.

## Discussion

Recent studies demonstrated mature hepatocytes serve as the major source for hepatocyte renewal and regeneration using genetic lineage tracing of mature hepatocytes in chronic liver injury^10–12,14–17^. Malato Y. et al. found newly formed hepatocytes derived from preexisting hepatocytes in the normal liver and acute injury; further, conversion of hepatocytes into BECs was not found in commonly used models of biliary injury^10^. Previous studies and our study demonstrated that hepatocytes undergo reversible ductal metaplasia in response to chronic injury^39,40^, expand and subsequently redifferentiate into functional hepatocytes^17^. The difference between Malato Y.’s study and our researches may be attributed to the time of liver injury and the type of liver injury. However, the mechanism underlying the conversion of mature hepatocytes into ductal BECs has not been investigated until now. Previous studies have demonstrated that LPC niches maintain the characteristics of LPCs and the balance between their activation, proliferation, and differentiation^18,24,26,36,41^. In this study, we demonstrated that MFBs derived from quiescent HSCs induced the conversion of mature hepatocytes into ductal BECs in mouse models of liver fibrosis. In addition, laminin-rich ECM secreted by MFBs induced hepatocyte-to-ductal metaplasia through integrin ɑvβ6 signal pathway. Thus the strategies to improve regeneration in cirrhotic liver should be developed to boost regeneration through modifying LPC niche in future.

The niche in which stem cells reside is the key element to regulate stem cell homeostasis. The Canals of Hering and bile ductules localized in the portal tract and the periportal parenchyma are believed to be the LPC niche. LPC niche is composed of MFBs, macrophages, and ECM in rodent models of severe liver injury and human disease^24,41^. In mouse model of CDE-induced chronic injury, HSC activation occurred prior to LPC expansion^26^. The depletion of MFBs by gliotoxin inhibited oval cell reaction which was revealed by expression of CK19^42^. All these indicated a fundamental role of MFBs during BECs activation. To determine the role of MFBs in BECs activation, animal models containing ductal BEC expansion and HSCs activation should be created. Mouse models of TAA, CCl_4_, DEN/CCl_4_-induced chronic injury were used in our study due to extensive HSC activation and a florid BEC response; whereas, extensive activation of HSCs was not observed in DDC and CDE-induced chronic injury. Importantly, animal models for manipulating HSC expression should be created in order to determine the contribution of MFBs to BEC activation, three models to manipulate HSC expression have been established to deplete HSCs in vivo, by using gliotoxin^42,43^, gliotoxin-coupled antibodies (Abs) against synaptophysin^44,45^ and transgenic mice expressing the herpes simplex virus thymidine kinase gene (HSV-Tk) driven by glial fibrillary acidic protein (GFAP) promoter^46^. However, gliotoxin has broad actions in vivo and in culture, targeting not only HSCs, but also immune and endothelial cells and hepatocytes. Previous studies showed that DAPT depleted MFBs specifically in vitro and in vivo^25,32^, thus DAPT was used to deplete MFBs in our study. Our study demonstrated DAPT inhibited HSC activation specifically, which was revealed by the markers of MFBs, macrophage (F4/80), endothelial cells (CD31), hepatocytes (HNF4ɑ), and T lymphocytes (CD4, CD8). This strategy for the depletion of MFBs was a sample method compared with transgenic mice^46^. In addition, in vitro data demonstrated DAPT had no effect on the expression of ductal BEC markers in chronic injured hepatocytes. Thus, DAPT was used to manipulate HSC expression in our study.

Cellular signaling between ductal BECs and the surrounding ECM is an important determinant of ductal BECs behavior^36^. In chronic liver injury, there is a requirement for collagen matrix to be degraded in order for ductal BECs to be activated and regenerate the liver^36^. Failure to degrade collagen-I critically impairs HSC apoptosis and prevent the effective restoration of hepatocyte mass in liver fibrosis^36,47^. In chronic injured liver, MFBs derived from quiescent HSCs produce laminin-rich ECM. Laminin–progenitor cell interactions within the LPC niche are critical for LPC-mediated regeneration^36^. Laminin is required to maintain LPCs in an undifferentiated biliary state^24^. Thus, we determined the role of laminin in the conversion of mature hepatocytes into ductal BECs. Interestingly, we found laminin secreted by MFBs promoted the conversion of mature hepatocytes into ductal BECs in vitro. Laminins are heterotrimeric proteins that contain an α-chain, a β-chain, and a γ-chain. Combinations of these chains give rise to 16 distinct isoforms, which are expressed in tissue-specific and developmentally regulated manners^48^. Thus, we speculate specific laminin isoforms are involved in the hepatocyte–cholangiocyte conversion. The identification of a specific laminin subtype involved in the hepatocyte–cholangiocyte conversion is an important issue. Thus, further studies should be carried out in future.

Integrin as its receptor is a transmembrane dimeric protein on the cell surface composed of noncovalently associated ɑ and β subunits, and facilitate cell–ECM adhesion. Upon ligand binding, integrins activate signal transduction pathways that mediate cellular signals, such as regulation of the cell cycle, organization of the intracellular cytoskeleton, and movement of new receptors to the cell membrane^49^. Integrin ɑvβ6 has an important role in models of fibrosis, such as lungs, liver, and kidney. Connective tissue growth factor (CTGF) and integrin ɑvβ6 regulate oval cell activation and fibrosis, probably through interacting with their common matrix and signal partners, fibronectin and TGF-β1^37^. Inhibition of integrin ɑvβ6 expression through genetic disruption or selective antibodies inhibits progenitor cell responses in mouse models of chronic biliary injury^38^. Thus, the involvement of integrin ɑvβ6 in hepatocyte-to-ductal metaplasia was determined in mouse models of liver fibrosis. In our study, up-regulation of integrin ɑvβ6 in ductal BECs was shown in fibrotic liver, and siRNA-mediated inhibition of integrin ɑvβ6 expression in chronic injured hepatocytes resulted in the suppression of hepatocyte-to-ductal metaplasia.

In summary, MFBs in niche of LPCs induce the conversion of chronic injured hepatocytes into ductal BECs through laminin–ɑvβ6 integrin, which reveals that the strategy improve liver regeneration in fibrotic liver through the modification of LPC niche in future.

## Supplementary information

Supplementary Figure Legends

Figure S1

Figure S2

Figure S3

Figure S4

Figure S5

Figure S6

Supplementary Table 1
