# Supplementary material for: Myofibroblast induces hepatocyte-to-ductal metaplasia via laminin–ɑvβ6 integrin in liver fibrosis
Source: Cell Death Dis. 2020 Mar 23;11(3):199. doi: 10.1038/s41419-020-2372-9 (PMC7090046; doi:10.1038/s41419-020-2372-9)
Supplement: Supplementary file 1 — Supplementary Figure Legends [file 41419_2020_2372_MOESM1_ESM.docx]

**Supplementary Information**

**Myoﬁbroblast Induces Hepatocyte-to-ductal Metaplasia via Laminin-ɑvβ6 Integrin in Liver Fibrosis**

Ting Xu ^1^•Zhiwen Lu ^1^•Zhuanglong Xiao ^1^• Fang Liu ^2^• Yuhua Chen ^1^• Zhijun Wang ^1^•Shenghua Zhu ^1^•Yuhu Song^1^

^1^Division of Gastroenterology, Union Hospital, Tongji Medical College, Huazhong University of Science and Technology, Wuhan 430022, China;

^2^Institute of Hematology, Union Hospital, Tongji Medical College, Huazhong University of Science and Technology, Wuhan 430022, China

*These authors contributed equally: Ting. Xu, Zhiwen. Lu, Zhuanglong. Xiao

**Corresponding author:**

Dr. Yuhu Song

Division of Gastroenterology, Union Hospital, Tongji Medical College, Huazhong University of Science and Technology,Wuhan 430022, China

**Email**: [yuhusong@yahoo.com](mailto:yuhusong@yahoo.com) or [yuhusong@163.com](mailto:yhsong@tjh.tjmu.edu.cn)

**Telephone:** 0086-27-85726678; **Fax:** 0086-27-85726057

**Running head:** Myoﬁbroblast in hepatocyte-to-ductal metaplasia

**Figure S1.** Inhibition of HSC activation diminished the expansion of ductal biliary epithelial cells (BECs) (quantitative analysis of Sirius red staining and immunohistochemical staining).

1. Quantitative assessment of collagen staining (Sirius red,100×) and HSC activation (ɑ-SMA staining, 200×);
2. quantitative assessment of ductal BECs expression (CK19, OPN and SOX9, 200×).

Each bar represents the mean ± S.D. for at least triplicate experiments and the p-value was determined by Student’s t-test.(*** P <0.001, ** P <0.01, * P<0.05).

**Figure S2.** DAPT inhibited the activation of hepatic stellate cells and had no effect on the expression of LPC markers in chronic injured hepatocytes in vitro.

1. Hepatic stellate cells were isolated from mouse models of liver fibrosis, and the purity of myofibroblasts(MFBs)/activated HSCs was demonstrated by immunofluorescence of ɑ-smooth muscle actin (ɑ-SMA), a marker of activated HSCs.
2. Myofibroblasts(MFBs) were treated with DAPT, and the result demonstrated the down-regulation of ɑ-SMA expression, which indicated that DAPT inhibited HSCs activation.
3. Chronic injured hepatocytes were isolated from mouse models of liver fibrosis, the purity of chronic injured hepatocytes was demonstrated by immunofluorescence of albumin.
4. Chronic injured hepatocytes were treated with DAPT, and the result demonstrated DAPT had no effect on the expression of BEC markers (CK19、SOX9、OPN) in chronic injured hepatocytes.

**Figure S3.** DAPT-mediated inhibition of HSC activation did not change the expression of endothelial cell (CD 31), macrophage (F4/80), HNF4ɑ, T lymphocytes (CD4, CD8) and caspase-3 in mouse models of liver fibrosis.

1. Experiment design for TAA-induced liver fibrosis or DAPT-mediated inhibition of HSC activation in vivo. Vertical lines represent weekly intraperitoneal injections of TAA or TAA/DAPT.
2. HE staining and immunohistochemical staining of fibrotic livers from TAA-treated mice treated by DAPT or the control. Immunohistochemical staining demonstrated that DAPT did not change the expression of endothelial cell (CD 31), macrophage (F4/80), HNF4ɑ, T lymphocytes (CD4, CD8) and caspase-3
3. Experiment design for CCl_4_-induced liver fibrosis or DAPT-mediated inhibition of HSC activation in vivo.
4. HE staining and immunohistochemical staining of fibrotic livers from CCl_4_-treated mice administrated with DAPT or the control. Immunohistochemical staining demonstrated that the expression of endothelial cell (CD 31), macrophage (F4/80), HNF4ɑ, T lymphocytes (CD4, CD8) and caspase-3 remained unchanged in DAPT-treated mice compared with the control.
5. Experiment design for DEN/CCl_4_-induced liver fibrosis or DAPT-mediated inhibition of HSC activation in vivo.
6. HE staining and immunohistochemical staining of fibrotic livers from DEN/CCl_4_-treated mice treated by DAPT or the control. Immunohistochemical staining demonstrated that DAPT did not change the expression of endothelial cell (CD 31) and macrophage (F4/80), T lymphocytes (CD4, CD8) and caspase-3.

Each bar represents the mean ± S.D. for at least triplicate experiments and the p-value was determined by Student’s t-test. (*** P <0.001, ** P <0.01, * P<0.05).

**Figure S4.** Immunoﬂuorescence staining demonstrated that myoﬁbroblasts (MFBs) induce the conversion of mature hepatocytes into ductal biliary epithelial cells in vivo

1. Immunostaining of ductal BECs marker (CK19) and myofibroblasts (aSMA)in fibrotic liver of Rosa^YFP^ mice which received AAV8-TBG-Cre; the result revealed neighborhood of myofibroblasts (MFBs) and hepatocytes-derived ductal BECs in mouse models of liver fibrosis.
2. Myoﬁbroblasts (MFBs) induce the conversion of mature hepatocytes into ductal biliary epithelial cells in vivo (quantitative assessment of ductal BECs expression).

Each bar represents the mean ± S.D. for at least triplicate experiments and the p-value was determined by Student’s t-test. (*** P <0.001, ** P <0.01, * P<0.05).

**Figure S5**. Myoﬁbroblasts(MFBs) induce the conversion of mature hepatocytes into ductal biliary epithelial cells in vitro.

1. Hepatocytes isolated from normal mice were co-cultured with myofibroblasts (MFBs); immunoﬂuorescence staining showed up-regulation of ductal BECs marker in hepatocytes.
2. Hepatocytes isolated from normal mice were treated with 2 mM CCl4, and then co-cultured with myoﬁbroblasts (MFBs); immunoﬂuorescence staining showed up-regulation of ductal BECs marker in hepatocytes.
3. Immunoﬂuorescence staining determined the expression of ductal BECs marker in chronic injured hepatocytes upon the treatment of myofibroblasts at different time.
4. Myoﬁbroblasts were treated with DAPT, and then co-cultured with chronic injured hepatocytes. Immunoﬂuorescence staining and immunoblotting analysis showed that DAPT-mediated inhibition of HSC activation resulted in an reduction in the expression of ductal BECs markers.

**Figure S6.** Myofibroblasts（MFBs） induce the conversion of mature hepatocytes into ductal biliary epithelial cells through the interaction of laminin-ɑvβ6 integrin.

1. ELISA analysis demonstrated the increase of laminin concentration in culture medium of activated HSCs compared with the control, and a decrease in laminin concentration upon DAPT-mediated inhibition of HSC activation. Each bar represents the mean ± S.D. for at least triplicate experiments and the p-value was determined by Student’s t-test. (*** P <0.001, ** P <0.01, * P<0.05).
2. Immunoblotting demonstrated up-regulation of ductal BECs marker (CK19, OPN and SOX9) and down-regulation of hepatocytes marker (HNF4ɑ) in chronic injured hepatocytes upon the treatment of laminin.
3. Up-regulation of integrin ɑvβ6 expression in periportal area was observed in fibrotic liver via immunohistochemical staining.
4. Immunoﬂuorescent confocal images of the liver demonstrated that BECs express integrin ɑvβ6 in mouse models of liver fibrosis. CK19 is a marker of BECs.
5. Chronic injured hepatocytes isolated from fibrotic liver were lysed, and then subjected to immunoprecipitation with preimmune sera, antibody to laminin; and followed by immunoblotted (IB) with laminin or integrin β6 antibodies. Coprecipitation of laminin-integrin β6 was demonstrated, which indicated that laminins exert their effect through binding integrins β6.
