# Supplementary figures and images for: Myofibroblast induces hepatocyte-to-ductal metaplasia via laminin–ɑvβ6 integrin in liver fibrosis
Source: Cell Death Dis. 2020 Mar 23;11(3):199. doi: 10.1038/s41419-020-2372-9 (PMC7090046; doi:10.1038/s41419-020-2372-9)

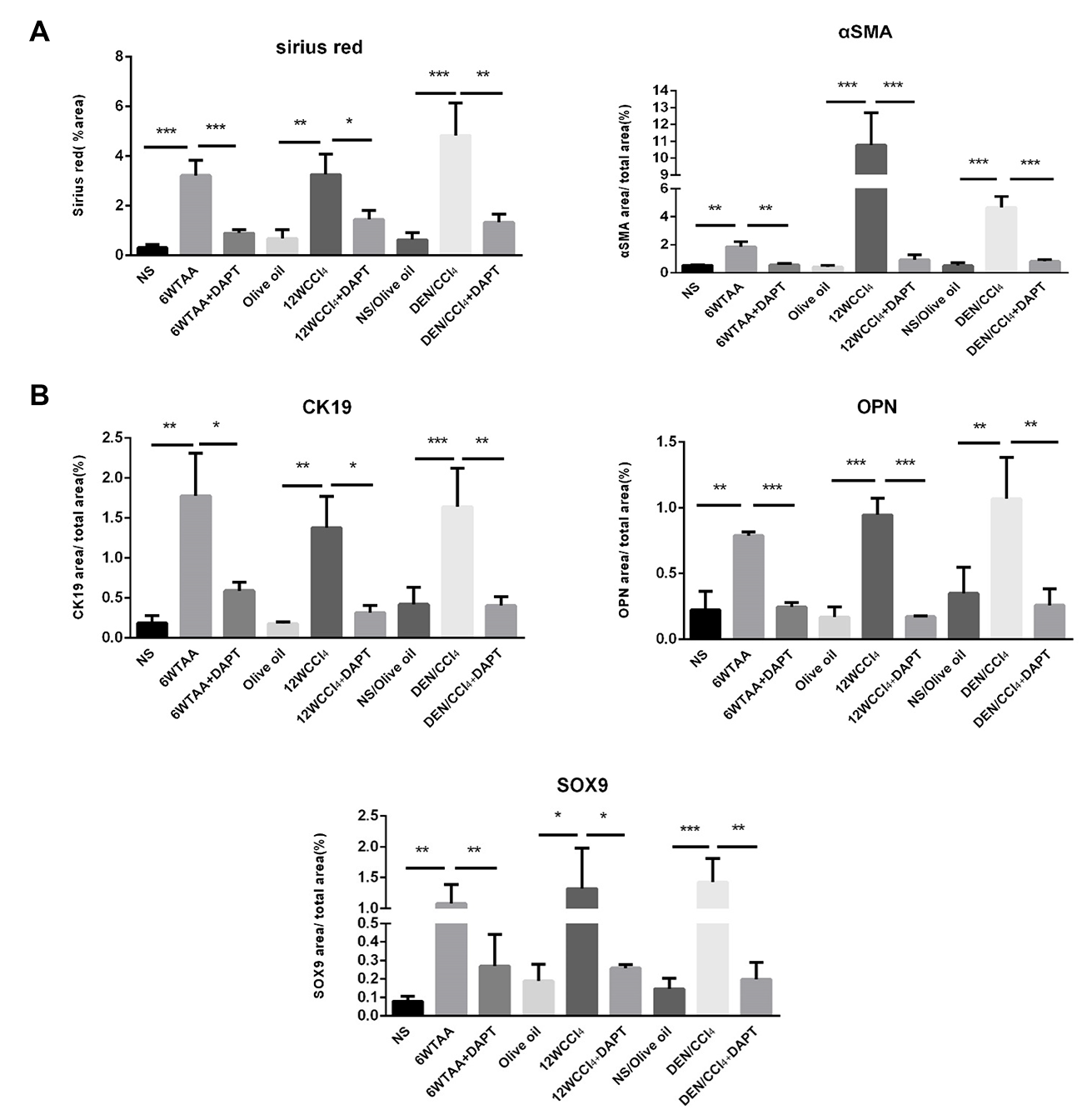

Supplement: Supplementary file 2 — Figure S1 [file 41419_2020_2372_MOESM2_ESM.png]

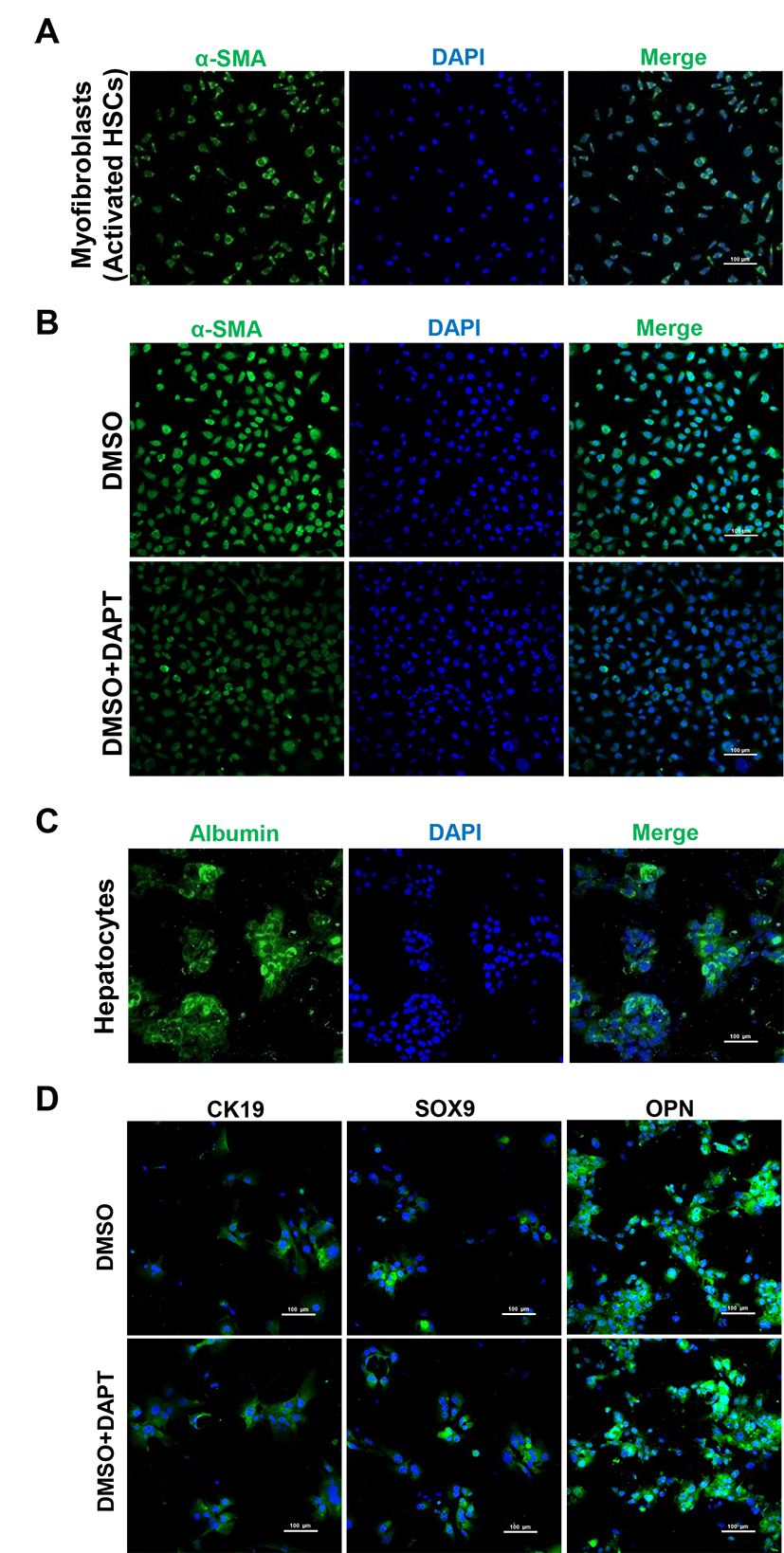

Supplement: Supplementary file 3 — Figure S2 [file 41419_2020_2372_MOESM3_ESM.png]

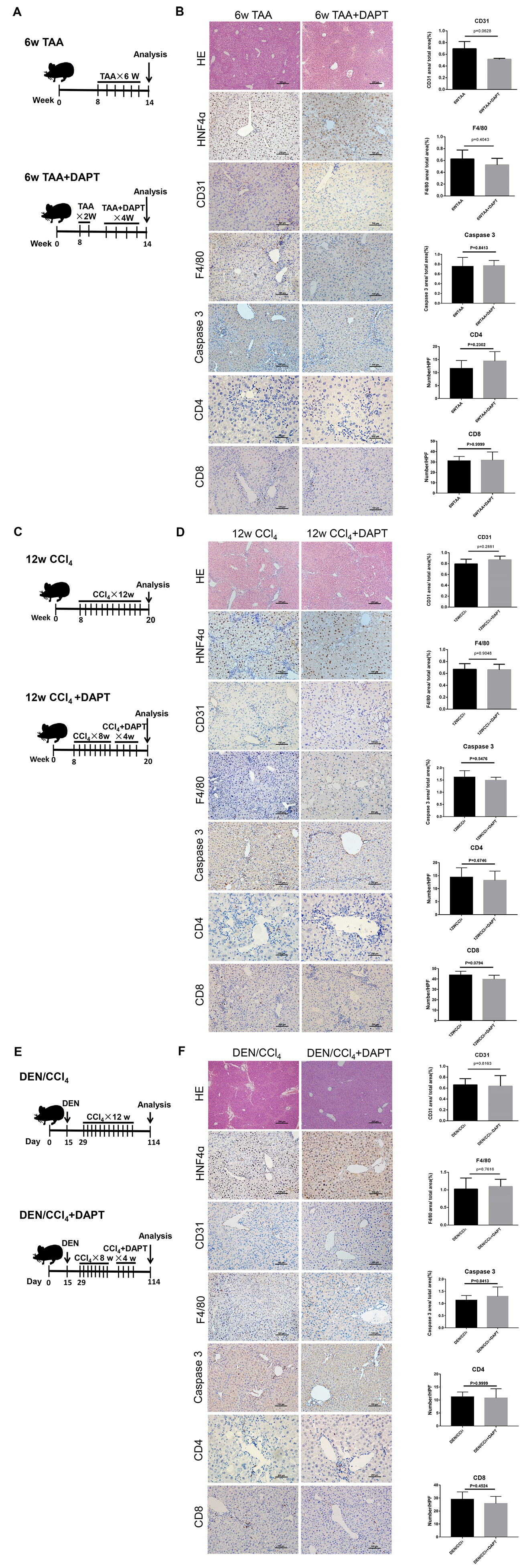

Supplement: Supplementary file 4 — Figure S3 [file 41419_2020_2372_MOESM4_ESM.png]

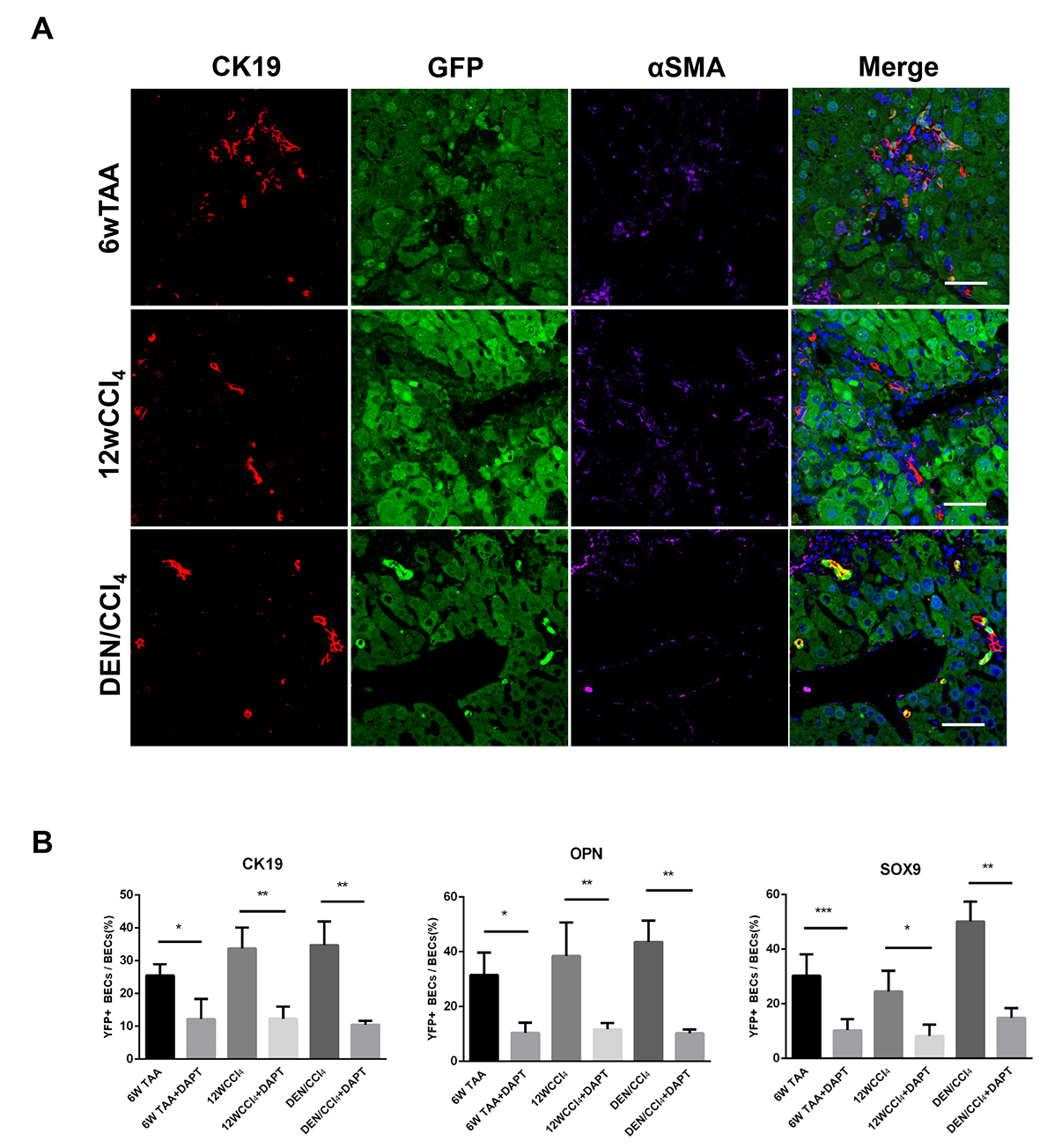

Supplement: Supplementary file 5 — Figure S4 [file 41419_2020_2372_MOESM5_ESM.png]

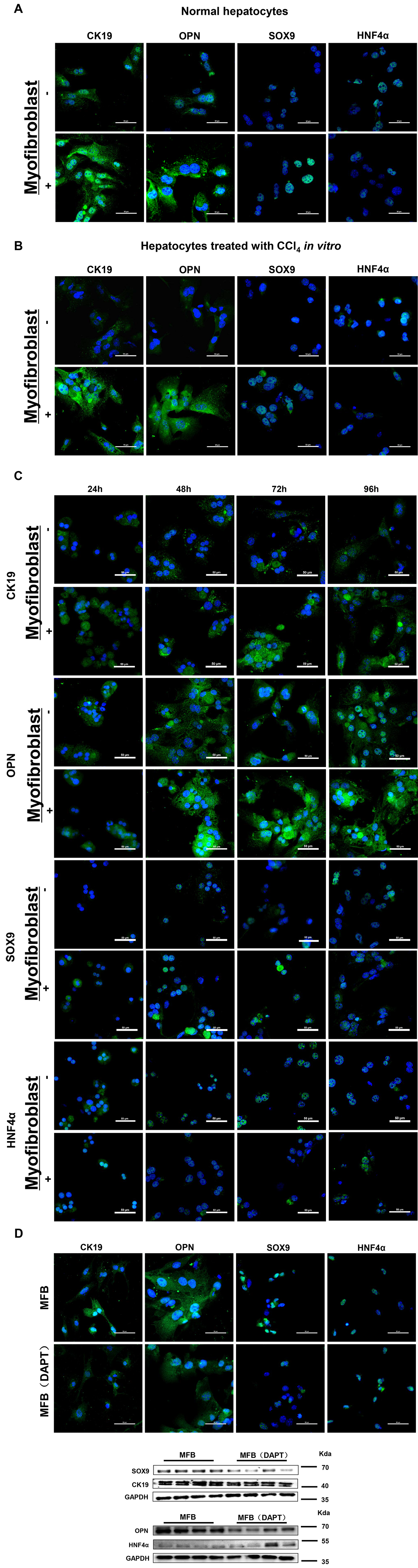

Supplement: Supplementary file 6 — Figure S5 [file 41419_2020_2372_MOESM6_ESM.png]

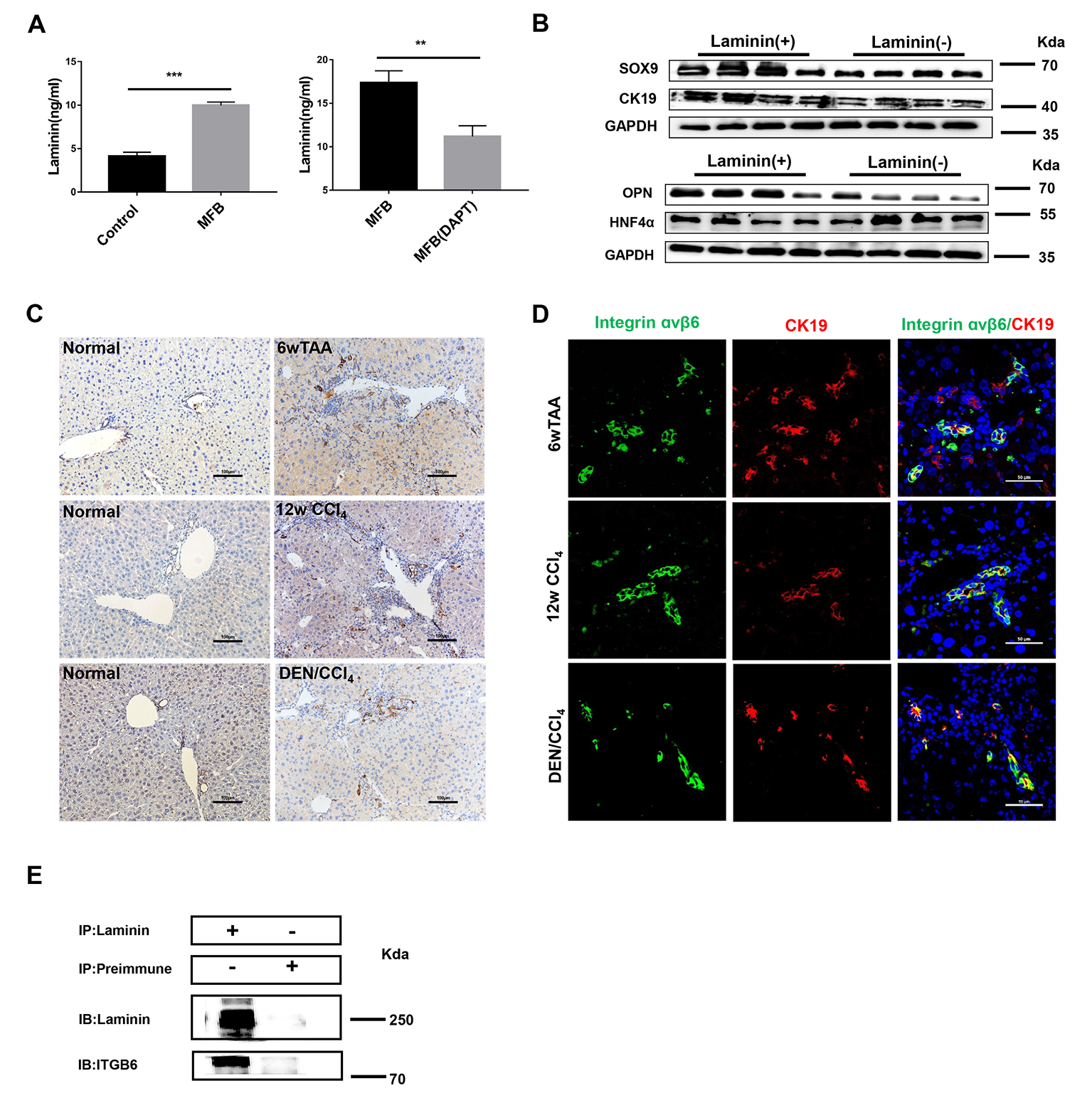

Supplement: Supplementary file 7 — Figure S6 [file 41419_2020_2372_MOESM7_ESM.png]
