# Supplementary material for: Myofibroblast induces hepatocyte-to-ductal metaplasia via laminin–ɑvβ6 integrin in liver fibrosis
Source: Cell Death Dis. 2020 Mar 23;11(3):199. doi: 10.1038/s41419-020-2372-9 (PMC7090046; doi:10.1038/s41419-020-2372-9)
Supplement: Supplementary file 8 — Supplementary Table 1 [file 41419_2020_2372_MOESM8_ESM.docx]

**Table 1:**

**Key resourse table**

| Reagents or resources | Source | Application | Catalogue number |
| --- | --- | --- | --- |
| Antibodies | | | |
| Rabbit anti- HNF4α | Abcam | IHC | ab201460 |
| Rabbit anti- CK19 | Proteintech | IHC、IF | 10712-1-AP |
| Rabbit anti- SOX9 | Millipore | IHC、IF | AB5535 |
| Goat anti- OPN | R&D systems | IHC、IF | AF808 |
| Rabbit anti- GFP | Proteintech | IF | 50430-2-AP |
| Rabbit anti- aSMA | Abcam | IHC、IF | ab5694 |
| Rabbit anti- CD31 | Abcam | IHC | ab28364 |
| Goat anti- GFP | Abcam | IF | ab6673 |
| Rat anti-F4/80 | Abcam | IHC | ab6640 |
| Goat anti- ITGB6 | R&D systems | IHC、IF | AF2389 |
| Rabbit anti- ITGB6 | ABclonal technology | WB | A16904 |
| Rabbit anti- Alb | Proteintech | IF | 16475-1-AP |
| Rabbit anti- HNF4α | ArigoBiolaboratories | IF | ARG-55328 |
| Rabbit anti-Laminin | Abcam | IP | ab11575 |
| Rat anti-MIC1-13 | Grompe Lab | Flow cytometry | Gift |
| APC Rat anti-mouse  MIC1-1C3 | BD PharMingen | Flow cytometry | NBP1-18961 |
| PE-Cy7 Rat anti-mouse CD11b | BD PharMingen | Flow cytometry | 552850 |
| PE-Cy7 Rat anti-mouse CD31 | BD PharMingen | Flow cytometry | 561410 |
| PE-Cy7 Rat anti-mouse CD45 | BD PharMingen | Flow cytometry | 552848 |
| Percp-Cyanine5.5 anti-mouse CD26 | eBioscience | Flow cytometry | H194-112 |
| Alexa Fluor 647 Goat anti Rat IgG (H+L) | Cell Signaling Technology | Flow cytometry | 4418S |
| Alexa Fluor 488 Donkey anti Rabbit IgG (H+L) | Antgene | IF | ANT024s |
| Alexa Fluor 594 Donkey anti Rabbit IgG(H+L) | Antgene | IF | ANT030s |
| Alexa Fluor 594 Donkey anti Rabbit IgG (H+L) | Life technologies | IF | A21207 |
| Alexa Fluor 488 Donkey anti goat IgG (H+L) | Antgene | IF | ANT025 |
| Alexa Fluor 594 Donkey anti goat IgG (H+L) | Antgene | IF | ANT031 |
| **Chemicals, Peptides, and Recombinant Proteins** | | | |
| Thioacetamide(TAA) | TCI | Mice model | T0187 |
| N-Nitrosodiethylamine(DEN) | TCI | Mice model | D0516 |
| Carbon tetrachloride | Makclin | Mice model | C822982 |
| Olive oil | Makclin | Solvent for CCl_4_ | O815211 |
| Corn oil | Sigma | Solvent for DMSO | C8267 |
| DAPT (GSI-IX) | Selleck&Bimake | Depletion of myofibroblast | S2215 |
| Dimethyl sulfoxide(DMSO) | MP Biomedicals | Solvent | 196055 |
| Laminin | Sigma | Cell culture | L2020 |
| Penicillin- Streptomycin | Sigma | Cell culture | V900929 |
| DMEM basic | Gibco | Cell culture | C11995500BT |
| Fetal Bovine Serum | Gibco | Cell culture | 10270-106 |
| Sirius red staining | Solarbio | ECM depostion | G1470-2 |
| DAPI | Sigma | IF | [28718-90-3](https://www.sigmaaldrich.com/catalog/search?term=28718-90-3&interface=CAS%20No.&N=0&mode=partialmax&lang=zh&region=CN&focus=product) |
| Propidium iodide | Sigma | Flow cytometry | P4170 |
| Trizol reagent | Invitrogen | RNA extraction | 15596-018 |
| RNAiso Plus | Takara | RNA extraction | 9109, |
| TB Green™ Premix Ex Taq™ | Takara | RT-PCR | RR420A |
| PrimeScript™ RT Master Mix | Takara | RT | RR036A |
| Collagenase Type IV | Gibco | HSC isolation | 9001-12-1 |
| DNase I | Roche | HSCisolation | 10104159001 |
| Lipofectamine^TM^2000 | Invitrogen | Cell transfection | 11668-019 |
| **Software and Algorithms** | | | |
| Adobe Photoshop CS6 | Adobe | Photo | Version19.1.2 |
| NIS-Elements Viewer | Laboratory Imaging | IF | Version3.20.02 |
| ImageJ | National Institutes of Health | IHC | Version1.51j8 |
| GraphPad Prism | GraphPad Software | Photo | Version5.0.1 |
| FlowJo | Becton, Dickinson & Company | Flow cytometry | Version10.0.7 |
